# Multiscale characterization and micromechanical modeling of crop stem materials

**DOI:** 10.1007/s10237-020-01369-6

**Published:** 2020-08-29

**Authors:** Tarun Gangwar, D. Jo Heuschele, George Annor, Alex Fok, Kevin P. Smith, Dominik Schillinger

**Affiliations:** 1 Department of Civil, Environmental, and Geo- Engineering, University of Minnesota, Twin Cities, USA; 2 Department of Agronomy and Plant Genetics, University of Minnesota, Twin Cities, USA; 3 Department of Food Science and Nutrition, University of Minnesota, Twin Cities, USA; 4 Minnesota Dental Research Center for Biomaterials and Biomechanics, University of Minnesota, Twin Cities, USA

**Keywords:** Continuum micromechanics, Microimaging, Hierarchical multiscale materials, Biomechanical tailoring, Oats

## Abstract

An essential prerequisite for the efficient biomechanical tailoring of crops is to accurately relate mechanical behavior to compositional and morphological properties across different length scales. In this article, we develop a multiscale approach to predict macroscale stiffness and strength properties of crop stem materials from their hierarchical microstructure. We first discuss the experimental multiscale characterization based on microimaging (micro-CT, light microscopy, transmission electron microscopy) and chemical analysis, with a particular focus on oat stems. We then derive in detail a general micromechanics-based model of macroscale stiffness and strength. We specify our model for oats and validate it against a series of bending experiments that we conducted with oat stem samples. In the context of biomechanical tailoring, we demonstrate that our model can predict the effects of genetic modifications of microscale composition and morphology on macroscale mechanical properties of thale cress that is available in the literature.

## Introduction

Recent advances in genomics have paved the way for biomechanical tailoring of crops (Brulé et al. 2016). The tailoring of crop properties could open up a plethora of agricultural and forestry applications. Examples are the optimized degradation of crop residues to biofuels (Vermerris and Abril 2015; McCann et al. 2014), breeding high yield and lodging resistant crop species (Berry et al. 2004), and the design of engineered plants with functional properties for sustainable construction (Schleicher et al. 2015; Holstov et al. 2015). Genetic alterations, however, can change the mechanical behavior of crops, with dire consequences on its growth progress and survival (Horvath et al. 2010; Koehler and Telewski 2006). Therefore, there is a growing interest in models that can accurately and consistently predict the mechanical behavior of the genetically altered crop plant structure.

**Fig. 1:**
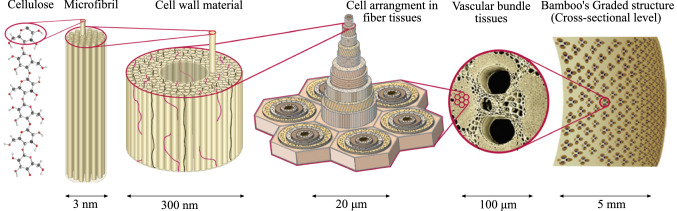
Hierarchical structure of bamboo. Adapted from Wegst et al. (2015) with kind permission from Nature Publishing Group

Plant materials organize themselves hierarchically across multiple length scales that range from base constituents such as lignin, cellulose, hemicellulose, and pectin to cell wall, functional tissue, organ, and whole plant levels (Wegst et al. 2015). Figure 1 illustrates the hierarchical organization for the case of bamboo. The stiffness and strength properties of crops thus depend on a complex combination of morphological and compositional parameters across these length scales. In addition to mechanical requirements, they must meet physiological and reproductive requirements for survival. These factors contribute to the growth and morphology of crops, and thus its mechanical properties. This complex interdependency makes it difficult to determine the contribution of each parameter to the mechanical properties through simple models. The detailed characterization of the hierarchical microstructure and a model considering all relevant length scales are therefore core prerequisites for the reliable prediction of crop mechanical properties.

The framework of continuum micromechanics (Zaoui 2002; Suquet 2014) presents a promising opportunity to model crop stem materials by rationally taking into account their hierarchical structure. On the one hand, the direct resolution of all scales in a numerical sense implies prohibitive computational cost (Nguyen et al. 2017; Yosibash et al. 2007), while other approaches such as the framework of cellular solids rely on extensive empirical parameter tuning to address the wide variability and complexity due to the multiscale nature of plants (Gibson and Ashby 1999; Gibson 2005). On the other hand, the success of stiffness and strength models based on continuum micromechanics has been established for hierarchical materials such as bone and cement in the last decade (Fritsch and Hellmich 2007; Fritsch et al. 2009; Pichler and Hellmich 2011; Hamed et al. 2012; Morin et al. 2017). In the plant modeling arena, continuum micromechanics approaches have been already used to predict microstructure–property relationships for wood (Hofstetter et al. 2005; Bader et al. 2010) and in our prior work on bamboo (Gangwar and Schillinger 2019).

In this article, we present a model for the stiffness and strength of crop stem materials in the framework of the cascade continuum micromechanics approach that was recently proposed for a broad range of inclusion morphologies (Timothy and Meschke 2016, 2017). In contrast to our work on bamboo that focused on functionally graded type materials, we focus here on the configuration of an inner layer of foam-like parenchyma cells surrounded by a dense outer shell, which is typical for crop stems (Gibson et al. 1995). This morphology brings along specific challenges for deriving microstructure–property relationships, which we describe and suggest solutions for.

For the example of oat, we experimentally profile the compositional and morphological properties across the hierarchical levels in the crop stem material, using microimaging technologies such as micro-CT, light microscopy, and transmission electron microscopy along with chemical composition analysis at the relevant scale. We show that all model parameters can be exclusively obtained from microimages without any phenomenological tuning. We assess the accuracy of our model predictions against flexural bending tests of oat stems. In addition, we demonstrate for thale cress that our model is able to consistently explain the contribution of compositional and morphological changes at different scales resulting from genetic mutation.

Our article is structured as follows: Sect. 2 characterizes the multiscale nature of crop stem material for the example of oat, using chemical analysis and microimaging technologies. In Sect. 3, we describe our micromechanics modeling approach for the stiffness and elastic limit strength of crop stem material and discuss essential modeling assumptions directly derived from plant physics. In Sect. 4, we validate model predictions that we obtained for parameters retrieved from the microimages reported in Sect. 2 with four-point bending flexural tests that we performed on oat stems. Finally, Sect. 5 demonstrates the ability of our model to quantify the effect of gene mutations and the associated compositional and morphological changes at multiple length scales on the macroscale mechanical properties.

**Fig. 2:**
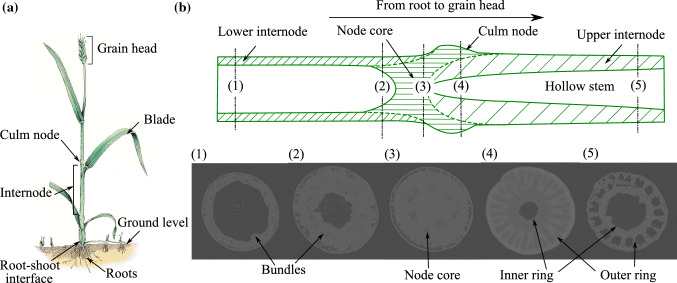
Typical anatomy of a crop plant and nodal region characterization with the help of micro-CT images

## Multiscale characterization of oat stem material

Crop stems usually consist of hollow and cylindrical internode regions separated by nodes, where leaves are attached. The length of the internodes increases from the ground to the top (root to grain head). Figure 2a illustrates a typical macroscale anatomy. An in-depth geometric and material characterization enables a better physics-based understanding of the mechanical behavior. In this section, we profile the compositional and morphological properties across scales for the example of the node and internode regions of oats. Advanced microimaging technologies such as computed tomography (CT), light microscopy, and transmission electron microscopy (TEM) along with chemical composition analysis enable the qualitative and quantitative description of various hierarchical levels in the plant stem organization. The oat stem specimens used for the analysis were grown at the University of Minnesota in greenhouses and fields in St. Paul, MN. Four commercial varieties of oats—Gopher, Reins, ND021052, and IL07-8721—were selected for the analysis. All specimens were checked for disease, pest damage, and other mechanical damage. Only specimens with no visible damage were included for the imaging and chemical analysis.

### Node morphology through micro-CT images

Figure 2b schematically describes the morphology of the node in the longitudinal direction, moving toward the grain head. This figure also shows the selected micro-CT images indicating their position in the node region. It is apparent from the micro-CT images that the morphology changes from left to right (moving upwards to the grain head) in the node region. Image 3 depicts the dense node core material, which is the hardest anatomical part of the oat stem. In images 4 and 5, the distinction between the outer and inner elliptical rings is apparent. In images 2 and 3, however, the elliptical rings are absent. This investigation reveals that the internode starts after the node core zone and the elliptical rings become smaller until they disappear, marking the start of the hollow upper internode. In conclusion, the structure of the node is asymmetrical along the length direction, which makes the node an important anatomical site in the context of the mechanical behavior of the plant. Similar morphological observations are reported for a wheat nodal region (Ghaffar and Fan 2015). In this contribution, however, we focus on the internode region.

**Fig. 3:**
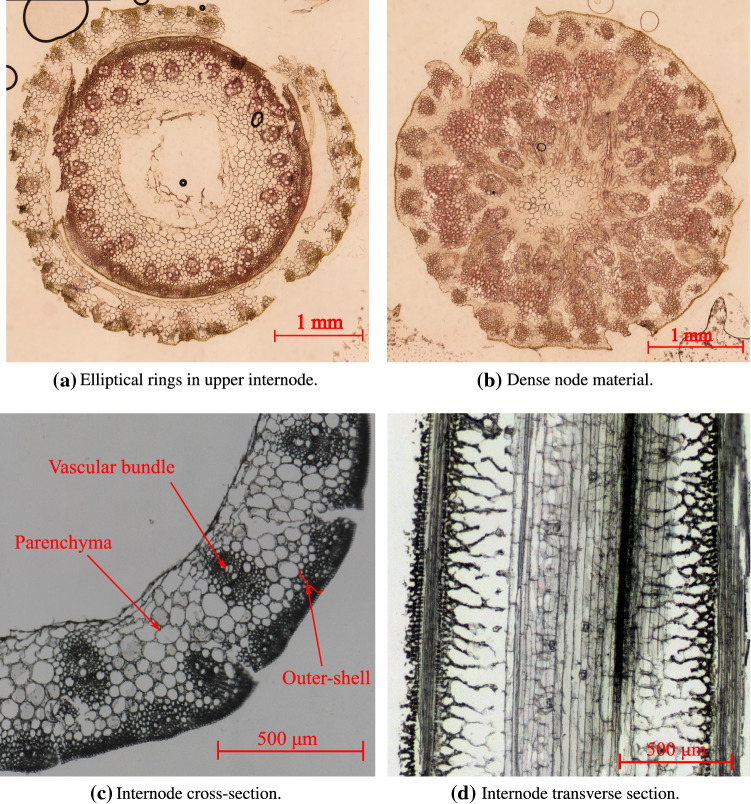
Cross-section profile of an oat stem through light microscopy images

### Cross-section through light microscopy images

Figure 3 illustrates anatomical details of the oat stem cross-section in different regions via light microscopy images. Figure 3a, b outlines the elliptical rings in the upper internode region and the dense node core material, respectively. Figure 3c, d shows the cross-section and the transverse-section of the oat stem internode region. The morphological longitudinal profile of the internode region is consistent, excluding the beginning and the termination stages of the internode. These images confirm the observations described in Sect. 2.1.

In Fig. 3c, d, dense epidermal layers with elongated collenchyma cell layers can be identified in the outer part of the internode cross-section. In this paper, we call these layers collectively the outer shell. The primary function of the outer shell is considered to stiffen the stem structure. In the inner part, vascular bundles are embedded in a matrix made up of parenchyma tissues. The vascular bundle tissues run through the length of the stem and have the main axis parallel to the longitudinal direction of the stem. The vascular bundles integrated with the parenchyma tissues are anticipated to act as a compliant core supporting the outer shell against loads beard during the lifetime of a plant.

### Functional regions through transmission electron microscopy images

**Fig. 4:**
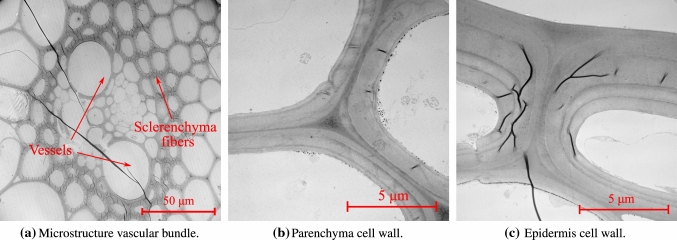
Functional region characterization through transmission electron microscopy (TEM)

Figure 4a demonstrates the morphology of fibers and vessels in a vascular bundle. Xylems and phloems are responsible for the transportation of nutrients and water into the plant. Xylem and phloem vessels are supported by sclerenchyma fiber sheath in a vascular bundle. The sclerenchyma fibers are long hollow tubes with thick cell walls oriented in the stem direction. The bundles are embedded in the parenchyma matrix. The parenchyma cells consist of thin cell walls and exhibit polyhedral geometry (see Fig. 4b). The cells are filled with living protoplasm and a major storage place for nutrients in the plant. The outer-shell region exhibits a similar morphology as sclerenchyma fibers. They have elongated thick cell walls surrounding holes (lumen) with a polygonic or circular cross-section (see Fig. 4c). The biological function of the epidermis is to control gas exchange and water balance.

The cell wall material in the functional regions is made up of cellulose, hemicellulose, and lignin. In the cell wall material, cellulose fibrils are helically wound with an average microfibril angle (MFA) to the cell axis that we denote by ${\bar{\theta }}$. Figure 4b, c depicts TEM images showing the multilayered cell wall structure in the parenchyma and epidermis cells in the oat stem, respectively. Lignin and hemicellulose are also hydrophilic sites within the cell wall material. Therefore, the properties of lignin and hemicellulose depend on moisture content (Cousins 1976, 1978).

**Table 1:** Chemical composition of the oat stem in percentage of total dry mass

Variety	Lignin	Hemicellulose	Cellulose
	$w_l$	$w_{hc}$	$w_c$
Gopher	50.69	18.80	30.51
Reins	65.30	15.28	19.42
ND021052	61.20	17.83	20.97
IL07-8721	62.47	18.24	19.29

### Chemical composition of the oat stem

Table 1 summarizes the chemical composition as the percentage of total dry mass in the oat stem. Field-grown plants were collected two weeks after flowering, and oven-dried for two days to preserve samples. Tissue samples were grounded and further divided into three types of chemical analysis. All chemicals used were research grade (Sigma-Aldrich, St. Louis, MO, USA). Hemicellulose concentration was determined by HPLC-MS (Anderson 2016; Anderson et al. 2015). Lignin was digested using a modified Klauson method to determine acid-soluble lignin (Aldaeus et al. 2011), while cellulose was determined by the one-step/two-step method (Yeats et al. 2016a, b).

In Table 2, we report the material constants of constituent materials cellulose, hemicellulose, lignin, and water. Native cellulose is a highly crystalline material that is assumed to exhibit a transversely isotropic behavior. This assumption has been confirmed experimentally in Matsuo et al. (1990), Nishino et al. (1995), where the longitudinal elastic properties of cellulose were determined by X-ray diffraction, and computationally in Mark (1967), Tashiro and Kobayashi (1996), where the full anisotropic behavior of cellulose was investigated via molecular dynamics simulations. Lignin and hemicellulose are hydrophilic amorphous materials. Cousins found that the stiffness of lignin and hemicellulose decreases with increasing moisture content (Cousins 1978, 1976). The living protoplasm of the cell is generally in a solute state. Therefore, from a mechanical point of view, it can be treated as water. We also assume that this water is in a drained state and therefore does not exert any pore pressure. Moreover, lignin is known to fail in shear, and its shear strength is reported as 20.2 MPa (Bader et al. 2010).

**Table 2:** Mass densities and stiffness properties of constituent phases

Constituent	Density	Material behavior	Elastic coefficients			References
Cellulose	1.59	Transversely isotropic	$E_{A} = 130$ $\nu _{A}= 0.087$	$E_{T} = 15$ $\nu _{T} = 0.49$	$G_{A} = 3$	Matsuo et al. (1990), Nishino et al. (1995), Mark (1967), Tashiro and Kobayashi (1996), Harrington et al. (1998)
Hemicellulose	1.50	Isotropic	$E = 9.00$	$\nu = 0.20$		Cousins (1978), Bergander and Salmén (2002), Persson (2000)
Lignin	1.37	Isotropic	$E = 5.25$	$\nu = 0.33$		Cousins (1976), Persson (2000)
Water	1	Isotropic	$k_w = 2.30$	$\mu _w = 0$		

$E_{A}$ ( $\nu _{A}$) and $E_T$ ($\nu _{T}$) denote axial and transverse stiffness moduli (Poisson’s ratios), $G_A$ denotes the axial shear modulus. *k* and $\mu$ denote bulk and shear moduli for isotropic material.

Values of *E*, *G*, *k* and $\mu$ are in GPa, density (g/cm$^3$) is obtained from de Borst and Bader (2014) and references therein.

## Multiscale modeling of stiffness and strength of crop stem material

In this section, we describe in detail our micromechanics-based modeling approach for crop stem materials. First, we review central concepts of continuum micromechanics, largely following the excellent presentations given in Zaoui (2002), Fritsch et al. (2009), Suquet (1997). We then describe the multistep micromechanical representation of the hierarchical organization of crop stem materials and establish microstructure–stiffness and microstructure–strength relationships. Our modeling decisions and required parameters are based on the multiscale characterization data discussed in the previous section.

### Conceptual overview of continuum micromechanics

#### Basic concepts and assumptions

The goal of continuum micromechanics is to replace the actual complex heterogeneous medium with a fictitious homogeneous one that has equivalent global behavior. An important objective is to establish an “equivalent homogeneous element” whose mechanical response is equivalent to a representative volume element (RVE) of the microheterogeneous material. One major prerequisite is that length scales are clearly separated:

1 $$\begin{aligned} d \ll l \ll L . \end{aligned}$$

Equation (1) states that the characteristic length scale of such an RVE, *l*, must be considerably larger than the dimensions of heterogeneities in the RVE, *d*. Moreover, *l* must be much smaller than the characteristic length scale of the variation in the loading on the macroscopic structure, *L*. In addition, the smallest characteristic length scale, *d*, should respect the lower bound on the length scale under which the assumptions of continuum mechanics are still valid.

In each phase *r*, the average microscopic stress $\varvec{\sigma }_r$, the average microscopic strain $\varvec{\varepsilon }_r$, and the phase stiffness ${{\mathbb{c}}}_r$ are related as

2 $$\begin{aligned} \varvec{\sigma }_r = {{\mathbb{c}}}_r :\varvec{\varepsilon }_r . \end{aligned}$$

Using the equilibrated microscopic stress field $\varvec{\sigma }$, that is, $\nabla \cdot \varvec{\sigma } = 0$, we can derive a homogenized macroscopic stress field $\varvec{{\varSigma }}$ by taking the average over the RVE volume *V* as

3 $$\begin{aligned} \varvec{{\varSigma }} = \langle \varvec{\sigma } \rangle = \frac{1}{V}\int _{V} \varvec{\sigma } \; dV = \sum _{r} \phi _r \varvec{\sigma }_r . \end{aligned}$$

Homogeneous strain boundary conditions at the boundary of the RVE can be parameterized as $u^g(x) = {\varvec{E}}x$, where ${\varvec{E}}$ is the macroscopic strain tensor, and *x* is the position vector at the boundary of the RVE. The resulting kinematically compatible microscopic strains $\varvec{\varepsilon }$ inside the RVE fulfill the volume-averaged condition as

4 $$\begin{aligned} {\varvec{E}} = \langle \varvec{\varepsilon } \rangle = \frac{1}{V}\int _{V} \varvec{\varepsilon } \; dV = \sum _{r} \phi _r \varvec{\varepsilon }_r . \end{aligned}$$

The homogenized macroscopic stress and strains, $\varvec{{\varSigma }}$ and ${\varvec{E}}$, are related to the homogenized macroscopic stiffness tensor ${\mathbb{C}}^{hom}$ as

5 $$\begin{aligned} \varvec{{\varSigma }} = {\mathbb{C}}^{hom} :{\varvec{E}}. \end{aligned}$$

The homogenized macroscopic stiffness tensor ${\mathbb{C}}^{hom}$ of the RVE needs to be linked with the geometric and mechanical characteristics of all constituent phases. A fourth-order concentration tensor ${\mathbb{A}}_r$ of phase *r* establishes the link between the average macroscopic strain ${\varvec{E}}$ and the average microscopic strain $\varvec{\varepsilon } _r$ in phase *r* as

6 $$\begin{aligned} \varvec{\varepsilon } _r = {\mathbb{A}}_r :{\varvec{E}}. \end{aligned}$$

Inserting (6) into (2) and averaging over all the phases according to (3) yields

7 $$\begin{aligned} \varvec{{\varSigma }} = \sum _{r} \phi _r {{\mathbb{c}}}_r: {\mathbb{A}}_r:{\varvec{E}}. \end{aligned}$$

From (7) and (5), we identify the relation between the macroscopic stiffness ${\mathbb{C}}^{hom}$, and the phase stiffnesses ${{\mathbb{c}}}_r$ and the concentration tensors ${\mathbb{A}}_r$ as

8 $$\begin{aligned} {\mathbb{C}}^{hom} = \sum _{r} \phi _r {{\mathbb{c}}}_r: {\mathbb{A}}_r. \end{aligned}$$

#### Eshelby’s analytical solution-based elastic homogenization

The computation of the concentration tensor ${\mathbb{A}}_r$ can be based on Eshelby-type analytical solutions. They relate the strain in an ellipsoidal inclusion perfectly bonded with the surrounded infinite matrix to the applied homogeneous strains at infinity. The elastic moduli of the matrix and the ellipsoidal inclusion are denoted as ${\mathbb{C}}^0$ and ${{\mathbb{c}}}_H$, respectively. Following (Zaoui 2002), the uniform strain field $\varvec{\varepsilon } _H$ in the inclusion in response to the homogeneous strain ${\varvec{E}}^0$ at infinity is

9 $$\begin{aligned} \varvec{\varepsilon } _H = [{\mathbb{I}} + {\mathbb{P}}_H^0:({{\mathbb{c}}}_H -{\mathbb{C}}^0)]^{-1}: {\varvec{E}}^0, \end{aligned}$$

where ${\mathbb{I}}$ is the fourth-order unit tensor and ${\mathbb{P}}_H^0$ is known as Hill tensor that characterizes the morphology of the inclusion and its interaction with the surrounding matrix. ${\mathbb{P}}_H^0$ depends on the morphology, that is, the shape and orientation of the inclusion as well as the stiffness tensor of the reference matrix ${\mathbb{C}}^0$. Analytical expressions for ${\mathbb{P}}_H^0$ can be found in Laws (1977, 1985).

For the estimation of ${\mathbb{A}}_r$, we approximate the average strains in each phase *r* by such inclusion strains, i.e., $\varvec{\varepsilon } _r = \varvec{\varepsilon } _H$. It implies that the average strains $\varvec{\varepsilon } _r$ in each phase of the RVE are considered to be equal to those of an ellipsoidal inhomogeneity with phase stiffness ${{\mathbb{c}}}_{r}$, embedded in a fictitious infinite matrix with stiffness ${\mathbb{C}}_{}^{0}$, subjected to some homogeneous strain ${\varvec{E}}^0$ applied at infinity. Using the strain average rule (4), we can find a relation between the homogenized macroscopic strain ${\varvec{E}}$ and the homogeneous strain ${\varvec{E}}^0$ at infinity in the fictitious matrix as

10 $$\begin{aligned} \begin{aligned} {\varvec{E}}^0 = \left\{ \sum _{r} \phi _r [{\mathbb{I}} + {\mathbb{P}}_{r}^{0}:({{\mathbb{c}}}_{r} - {\mathbb{C}}_{}^{0})]^{-1}\right\} ^{-1} : {\varvec{E}}. \end{aligned} \end{aligned}$$

The relation between ${\varvec{E}}^0$ and ${\varvec{E}}$ accounts for the influence of the fictitious matrix phase strains on the inclusion strains. The simplest case with ${\varvec{E}}^0 = {\varvec{E}}$ results in the dilute scheme, where inclusions do not affect each other. Here, we briefly summarize schemes that account for the influence on one phase caused by other phases. With $\varvec{\varepsilon } _r = \varvec{\varepsilon } _H$, substitution of ${\varvec{E}}^0$ in (9) and comparison with (6) yields the following estimate of the concentration strain tensor ${\mathbb{A}}_r$:

11 $$\begin{aligned} \begin{aligned} {\mathbb{A}}_r = [{\mathbb{I}} + {\mathbb{P}}_r^0:&({{\mathbb{c}}}_r -{\mathbb{C}}^0)]^{-1}: \\&\quad \left\{ \sum _{r} \phi _r [{\mathbb{I}} + {\mathbb{P}}_{r}^{0}:({{\mathbb{c}}}_{r} - {\mathbb{C}}_{}^{0})]^{-1}\right\} ^{-1}. \end{aligned} \end{aligned}$$

The expression for the homogenized stiffness ${\mathbb{C}}^{hom}$ follows from (11) and (8) as

12 $$\begin{aligned} \begin{aligned} {\mathbb{C}}^{hom} = \sum _{r} \phi _r &{{\mathbb{c}}}_r :[{\mathbb{I}} + {\mathbb{P}}_r^0:({{\mathbb{c}}}_r - {\mathbb{C}}^0)]^{-1}: \\&\quad \left[ \sum _{s} \phi _s [{\mathbb{I}} + {\mathbb{P}}_s^0:({{\mathbb{c}}}_s-{\mathbb{C}}^0)]^{-1}\right] ^{-1}. \end{aligned} \end{aligned}$$

In (11) and (12), ${\mathbb{C}}^0$ accounts for the influence that inclusions have on each other in the RVE. If one of the phases assumes the role of the matrix for other phases, that is, ${\mathbb{C}}^0 = {{\mathbb{c}}}_M$, where ${{\mathbb{c}}}_M$ represents the stiffness of the matrix phase, the homogenization method is called Mori-Tanaka scheme (Mori and Tanaka 1973; Wakashima and Tsukamoto 1991). Another way to capture this influence is averaging the response of all phases in the sense of a virtual matrix material, that is, ${\mathbb{C}}^0 = {\mathbb{C}}^{hom}$. This homogenization method is known as the self-consistent scheme (Kröner 1958; Hill 1963). It is particularly useful in the morphologically disordered case, for instance, when several phases are present that interpenetrate each other such that a clear distinction between matrix and inclusions is impossible.

#### Estimate of homogenized elastic limit strength

A macroscale RVE reaches the elastic limit state when any one of the constituents in the RVE yields. Let us focus on the weakest constituent phase, denoted by index $r = w$. We assume that its elastic limit behavior is described by the folllowing failure criterion

13 $$\begin{aligned} \varvec{{\mathfrak {f}}}_{w} (\varvec{\sigma }_w) \le 0, \end{aligned}$$

where $\varvec{\sigma }_w$ is the stress distribution in the weak phase *w*. We write $\varvec{\sigma }_w$ in terms of the effective strain $\varvec{\varepsilon }^{*}_w$ with the help of the elasticity tensor ${{\mathbb{c}}}_w$ as

14 $$\begin{aligned} \varvec{\sigma }_w = {{\mathbb{c}}}_w : \varvec{\varepsilon }^{*}_w. \end{aligned}$$

A natural choice for the effective strain tensor $\varvec{\varepsilon }^{*}_w$ would be the average phase strain introduced in (6). However, microscopic failure is governed by “peak strains” rather than by “average strains.” The strain peaks in phase *w* can be estimated with the second-order moment of the strain field in this phase, which is the quadratic strain average $\overline{\overline{\varvec{\varepsilon }}}_w$ over the phase volume $V_w$ expressed as

15 $$\begin{aligned} \varvec{\varepsilon }^{*}_w = \overline{\overline{\varvec{\varepsilon }}}_w = \langle \varvec{\varepsilon }:\varvec{\varepsilon } \rangle ^{1/2}_w = \left( \frac{1}{V_w} \int _{V_w}^{} \frac{1}{2} \varvec{\varepsilon }:\varvec{\varepsilon } dV \right) ^{1/2}. \end{aligned}$$

The stress tensor $\varvec{\sigma }_w$ can be computed in the weak phase with the effective strain $\varvec{\varepsilon }^{*}_w$, which allows us to evaluate the failure criterion for the weak phase from (13). ${\mathbb{C}}^{hom}$ represents the overall stiffness of the RVE as a function of the elastic stiffness coefficients of the individual constituent phases. The elastic coefficients, the bulk modulus and the shear modulus of the weak phase with the volume fraction ${\overline{\phi }}_w$ are denoted by $k_w$ and $\mu _{w}$, respectively. Following (Suquet 1997), the von Mises equivalent strain of the quadratic strain average $\overline{\overline{\varvec{\varepsilon }}}_w$ can be related to the macroscopic strains ${\varvec{E}}$ imposed on the boundary of the RVE as

16 $$\begin{aligned} \langle {\varepsilon }^2_{eq(w)} \rangle = \frac{1}{3{\overline{\phi }}_w} {\varvec{E}}: \frac{\partial \; {\mathbb{C}}^{hom}}{\partial \mu _{w}}:{\varvec{E}} . \end{aligned}$$

The von Mises equivalent stress $\sigma _{eq}$ is defined as

17 $$\begin{aligned} \sigma _{eq} = \left( \frac{3}{2}\sigma ^{dev}_{ij}\sigma ^{dev}_{ij}\right) ^{1/2}, \end{aligned}$$

where $\sigma ^{dev}_{ij}$ represents the deviatoric stress field. Following (16) and (17), the von Mises equivalent stress in the weak phase $\sigma _{eq(w)}$ is

18 $$\begin{aligned} \sigma _{eq(w)} = 3 \mu _w \varepsilon _{eq(w)} = \mu _w \left( \frac{1}{\phi _w} {\varvec{E}}: \frac{\partial {\mathbb{C}}^{hom}}{\partial \mu _{w}}:{\varvec{E}}\right) ^{1/2} . \end{aligned}$$

If $\varvec{{\mathfrak {f}}}_w$ is a scalar deviatoric stress-based failure criterion such as the von Mises criterion, then it can be expressed in terms of $\sigma _{eq(w)}$. It implies that the local phase-related failure criterion (13) can be expressed in terms of the macroscopic strains ${\varvec{E}}$ following (18). With ${\varvec{E}} = [{\mathbb{C}}^{hom}]^{-1}:\varvec{{\varSigma }}$, the weak phase criterion $\varvec{{\mathfrak {f}}}_w$ translates to the macroscopic failure criterion ${\mathfrak {F}}$ as

19 $$\begin{aligned} {\mathfrak {F}} (\varvec{{\varSigma }}) \le 0 . \end{aligned}$$

We note that the limiting stress level $\varvec{{\varSigma }}$ in (19) is the elastic limit strength of the RVE.

### From hierarchical representation to multistep micromechanics modeling

**Fig. 5:**
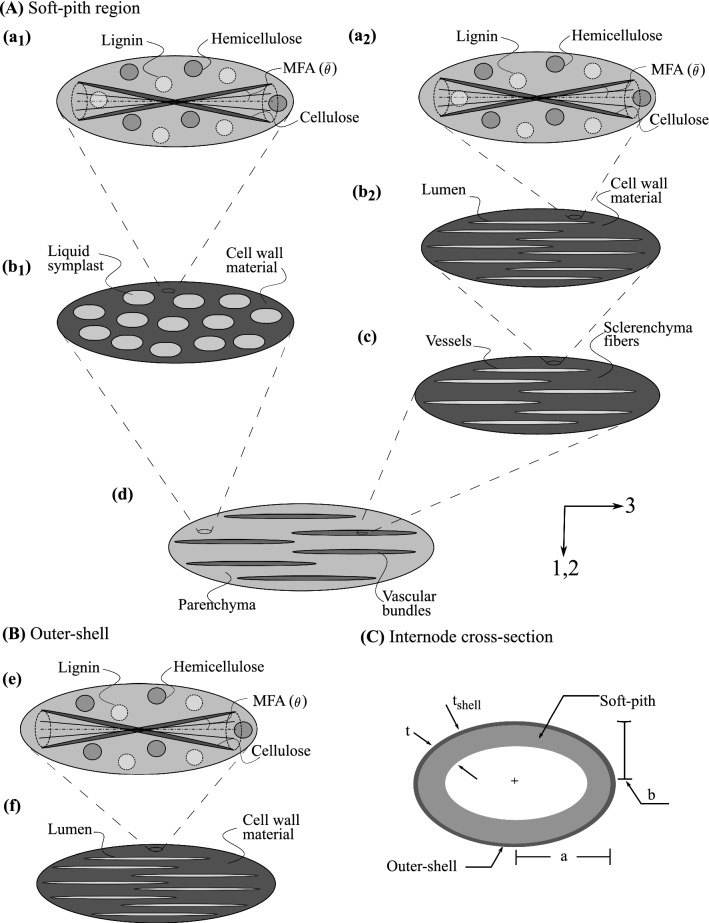
Multistep micromechanical representation of crop stem material. The two columns in **a** represent individual models for the parenchyma and vascular bundles in the soft-pith region. **b** depicts the multistep model for the outer-shell region and **c** a schematic representation of the stem cross-section

Figure 5 provides a summary of our multistep micromechanics model. Following Sect. 2, we transfer the multiscale characterization (given here for the example of an oat stem) in a multistep micromechanical representation. The cross-section of a crop stem consists of an outer shell and the inner foam-like soft pith. To this end, we build multistep micromechanics models for both regions independently and combine them together in the crop stem cross-section (see Fig. 5c). Here, we describe each RVE of the multistep model for outer-shell and soft-pith regions.

- At the finest scale in the soft-pith, we consider RVEs of the *cell wall material* in the parenchyma and sclerenchyma region, represented by level $(a_1)$ and $(a_2)$ in Fig. 5. Each RVE is regarded as a three-phase material, consisting of crystalline cellulose, hemicellulose, and lignin. The cylindrical cellulose fibrils helically wind around the lumen within the cell wall with an average inclination angle to the cell axis denoted as microfibril angle (MFA) ${\bar{\theta }}$. Since all three phases have contact with each other, we assume that they form an “average” transversely isotropic matrix. The “average” matrix hosts spherical inclusions of hemicellulose and lignin, and helically wound cylindrical inclusions of the cellulose fibrils.
- The RVE at level $(b_1)$ represents the *parenchyma* base tissues in the soft-pith region. It contains two phases: the cell wall material and the living symplast. The mechanical properties of living symplast are assumed equivalent to water. Due to its polyhedral geometry, we assume that the living symplast forms spheroidal inclusions in the matrix of the cell wall material.
- The RVE at level $(b_2)$ represents a *sclerenchyma fiber*, that consists of a matrix of cell wall material hosting cylindrical inclusions of lumens. The RVE at level (*c*) represents a *vascular bundle* in soft pith, where xylem and phloem tissues are surrounded by sclerenchyma sheath. The constituent phases of this RVE are sclerenchyma fibers (matrix) and vessels (cylindrical inclusion assumed to be filled with water).
- At the RVE level (*d*), the parenchyma base material and the vascular bundles are brought together to form *soft pith*. The vascular bundles are distributed in the parenchyma matrix. They run through the whole length of the stem and are modeled as cylindrical inclusions.
- The multistep model for the outer shell consists of two RVEs (see Fig. 5b). The RVE at level (*e*) represents a *cell wall material* for the outer-shell region. It is equivalent to the level $(a_2)$ in the soft-pith region. Level (*e*) forms matrix and hosts cylindrical inclusions of lumens for the RVE of an *outer-shell* material at level (*f*).

### Microstructure–stiffness relationship in the elastic range

We describe the homogenization procedure for the elastic stiffness coefficients for the example of oat. To this end, we consider the hierarchical structure in terms of the RVEs illustrated in Fig. 5. Homogenization in the elastic range is mainly based on the central relation (12).

#### Cell wall materials

In the RVE that corresponds to the *cell wall material* in the parenchyma region (level $a_1$ in Fig. 5), we denote the volume fractions of hemicellulose, lignin, and crystalline cellulose as $\phi _h^{wall,par}$, $\phi _l^{wall,par}$, and $\phi _{cc}^{wall,par}$, which satisfy $\phi _l^{wall,par} + \phi _h^{wall,par} + \phi _{cc}^{wall,par} = 1$. The stiffness tensors of hemicellulose, lignin, and cellulose, ${{\mathbb{c}}}_h$, ${{\mathbb{c}}}_l$, and ${{\mathbb{c}}}_{cc}$, can be filled with values from Table 2. All three phases are in contact with each other and form an intimate mixture. Therefore, we assume the self-consistent scheme with an “average” host matrix for this RVE. As discussed in the previous section, helically wound cylindrical inclusions of cellulose fibrils, and spherical inclusions of hemicellulose and lignin are embedded in this “average” matrix.

To account for the helical orientation of the fibrils, we assume that there are infinite cylindrical cellulose fibrils embedded in the “average” matrix in the RVE of the cell wall material (Hofstetter et al. 2006). The orientation of these inclusions is defined by the two angles ($\varphi , {\bar{\theta }}$). We can obtain the Hill tensor ${\mathbb{P}}_r^0 = {\mathbb{P}}_{cyl}^{wall,par}$ for a cylindrical inclusion in the transversely isotropic “average” matrix that refers to a local coordinate system (see Appendix A.1 in Fritsch et al. (2009)). Tensors ${{\mathbb{c}}}_{cc}(\varphi ,{\bar{\theta }})$ and ${\mathbb{P}}_{cyl}^{hl,par}(\varphi ,{\bar{\theta }})$ for one such cylindrical inclusion in the global coordinate system can be obtained by standard tensor transformations (Salençon 2012; Bao 2005).

The Hill tensor ${\mathbb{P}}_{sph}^{wall,par}$ describes the morphological distribution for the spherical inclusions of the hemicellulose and lignin in the RVE. For the calculation of its components, we refer to Appendix A. 2 in Fritsch et al. (2009). Following (12) with ${\mathbb{C}}^0 = {\mathbb{C}}^{hom} = {\mathbb{C}}_{wall}^{par}$, we arrive at the stiffness tensor ${\mathbb{C}}_{wall}^{par}$ of the cell wall material in the parenchyma region as

20 $$\begin{aligned} \begin{aligned} {\mathbb{C}}_{wall}^{par}&= \left\{ \sum _{r} \phi _r^{wall,par} {{\mathbb{c}}}_r:[{\mathbb{I}} + {\mathbb{P}}_{sph}^{wall,par} :({{\mathbb{c}}}_r - {\mathbb{C}}_{wall}^{par})]^{-1} \right. \\&\left. \qquad + \, \phi _{cc}^{wall,par}\big \langle {{\mathbb{c}}}_{cc}(\varphi ,{\bar{\theta }}): [{\mathbb{I}} + {\mathbb{P}}_{cyl}^{wall,par}(\varphi ,{\bar{\theta }}):\right. \\&\left. \qquad ({{\mathbb{c}}}_{cc}(\varphi ,{\bar{\theta }}) - {\mathbb{C}}_{wall}^{par})]^{-1}\big \rangle \right\} :\\&\quad \left\{ \sum _{s} \phi _s^{wall,par} [{\mathbb{I}} \; + {\mathbb{P}}_{sph}^{wall,par}: ({{\mathbb{c}}}_s - \; {\mathbb{C}}_{wall}^{par})]^{-1} \right. \\&\left. \qquad + \phi _{cc}^{wall,par}\big \langle [{\mathbb{I}} +{\mathbb{P}}_{cyl}^{wall,par}(\varphi ,{\bar{\theta }}):\right. \\&\left. \qquad ({{\mathbb{c}}}_{cc}(\varphi ,{\bar{\theta }}) - {\mathbb{C}}_{wall}^{par})]^{-1}\big \rangle \right\} ^{-1} ; \; r,s \in [h,l] \end{aligned} \end{aligned}$$

where the operator $\langle . \rangle$ is defined as

21 $$\begin{aligned} \langle g(\varphi ) \rangle = \frac{1}{2\pi } \int _{0}^{2\pi }{g(\varphi )d\varphi } . \end{aligned}$$

Due to its implicit format with respect to ${\mathbb{C}}_{wall}^{par}$, relation (20) is computed iteratively (Hellmich et al. 2004; Hellmich and Ulm 2002). We note that the integration over $\varphi$ in (21) is performed numerically with a simple rectangle rule.

We deal with the RVE of the *cell wall material* in the sclerenchyma region (level $a_2$ in Fig. 5) in a similar fashion, where the volume fraction of hemicellulose, lignin, and cellulose are $\phi _l^{wall,fib}$, $\phi _h^{wall,fib}$, and $\phi _{cc}^{wall,fib}$. The corresponding homogenized stiffness tensor ${\mathbb{C}}_{wall}^{fib}$ of the RVE is

22 $$\begin{aligned} \begin{aligned} {\mathbb{C}}_{wall}^{fib}&= \left\{ \sum _{r} \phi _r^{wall,fib} {{\mathbb{c}}}_r:[{\mathbb{I}} + {\mathbb{P}}_{sph}^{wall,fib} :({{\mathbb{c}}}_r - {\mathbb{C}}_{wall}^{fib})]^{-1} \right. \\&\left. \qquad + \phi _{cc}^{wall,fib}\big \langle {{\mathbb{c}}}_{cc}(\varphi ,{\bar{\theta }}): \right. \\&\left. \qquad [{\mathbb{I}} + {\mathbb{P}}_{cyl}^{wall,fib}(\varphi , {\bar{\theta }}):({{\mathbb{c}}}_{cc}(\varphi ,{\bar{\theta }}) \right. \\&\left. \qquad - {\mathbb{C}}_{wall}^{fib})]^{-1}\big \rangle \right\} : \\&\quad \left\{ \sum _{s} \phi _s^{wall,fib} [{\mathbb{I}} + {\mathbb{P}}_{sph}^{wall,fib}: ({{\mathbb{c}}}_s \right. \\&\left. \qquad - {\mathbb{C}}_{wall}^{fib})]^{-1} + \phi _{cc}^{wall,fib}\big \langle [{\mathbb{I}} + {\mathbb{P}}_{cyl}^{wall,fib}(\varphi ,{\bar{\theta }}):\right. \\&\left. \qquad ({{\mathbb{c}}}_{cc}(\varphi ,{\bar{\theta }}) - {\mathbb{C}}_{wall}^{fib})]^{-1}\big \rangle \right\} ^{-1} ; \; r,s \in [h,l] . \end{aligned} \end{aligned}$$

#### Parenchyma region

The RVE of the parenchyma (level $b_1$ in Fig. 5) contains the inclusions of the living symplast in the matrix of the cell wall material. Previous studies indicate that their volume fraction in the parenchyma is in the range of $0.5-0.8$ (Gangwar and Schillinger 2019). Neither the Mori-Tanaka scheme nor the self-consistent scheme is suitable at higher inclusion volume fractions. On the one hand, the Mori-Tanaka scheme assumes inclusions to be completely isolated with a continuous matrix, and this completely connected matrix contributes to the overall stiffness even at a very high volume fraction of the inclusions. Therefore, it largely overestimates the elastic properties at higher inclusion volume fractions. On the other hand, the self-consistent scheme assumes phases to be perfectly in contact with each other. Beyond a certain volume fraction of inclusions, however, the matrix does not have a sufficient volume fraction to form a connected matrix to resist material failure. Therefore, the self-consistent scheme leads to physically meaningless homogenization estimates above a particular inclusion volume fraction (Willis 1977).

Timothy and Meschke proposed the cascade continuum micromechanics (CCM) model to estimate elastic properties for a broad range of inclusion volume fractions (Timothy and Meschke 2016, 2017). We briefly outline the model in Appendix A for completeness, but refer interested readers to Timothy and Meschke (2016, (2017) for details. Basically, CCM is a set of matrix-inclusion problems obtained through recursion. Recursion or cascade level *n* represents the degree of connectivity of the inclusion phase (see 40). Following (Timothy and Meschke 2016), the morphology of the parenchyma region is identical to foam, and cascade level $n = 2$ leads to good agreement with experimental results. We use the CCM model with $n = 2$ to estimate the homogenized stiffness of the parenchyma region following (40) and (22).

We denote the volume fraction of the living symplast inclusions and the cell wall material matrix as $\phi _{ls}^{par}$ and $\phi _{wall}^{par}$, which satisfy $\phi _{ls}^{par} + \phi _{wall}^{par} = 1$. The elastic properties of the living symplast can be assumed to be equivalent to water, that is, ${{\mathbb{c}}}_w$. At cascade level $n = 1$, the cell wall material acts as a matrix, that is, ${{\mathbb{c}}}_m = {\mathbb{C}}^{par}_{wall}$ in (41a), in which the symplast forms spheroidal inclusions. With the Hill tensor ${\mathbb{P}}_r^0 = {\mathbb{P}}_{sphrd}^{wall}$, which corresponds to spheroidal inclusions with a known elongation ratio in the transversely isotropic matrix of the cell wall material (Laws 1985), the homogenized stiffness tensor ${\mathbb{C}}^{(1)}_{par}$ at cascade level $n = 1$ can be expressed as

23 $$\begin{aligned} \begin{aligned} {\mathbb{C}}_{par}^{(1)}= \Big \{&\phi _{wall}^{par} {\mathbb{C}}_{wall}^{par} + \phi _{ls}^{par} {{\mathbb{c}}}_w:[{\mathbb{I}} + {\mathbb{P}}_{sphrd}^{ wall}: \\& ({{\mathbb{c}}}_w - {\mathbb{C}}_{wall}^{par})]^{-1} \Big \} : \Big \{ \phi _{wall}^{par} {\mathbb{I}} + \phi _{ls}^{par}\, [{\mathbb{I}} \;+ \\& {\mathbb{P}}_{sphrd}^{ wall}: ({{\mathbb{c}}}_w - {\mathbb{C}}_{wall}^{par})]^{-1} \Big \}^{-1}. \end{aligned} \end{aligned}$$

The homogenized stiffness tensor ${\mathbb{C}}^{(1)}_{par}$ acts as a matrix for cascade level $n =2$. Following (41b) with analogous notations, we obtain the stiffness tensor of the parenchyma ${\mathbb{C}}_{par}$ as

24 $$\begin{aligned} \begin{aligned} {\mathbb{C}}_{par}^{}= \Big \{&\phi _{wall}^{par} {\mathbb{C}}_{par}^{(1)} + \phi _{ls}^{par} {{\mathbb{c}}}_w:[{\mathbb{I}} + {\mathbb{P}}_{sphrd}^{par,(1)}:\\& ({{\mathbb{c}}}_w - {\mathbb{C}}_{par}^{(1)})]^{-1} \Big \} :\Big \{ \phi _{wall}^{par} {\mathbb{I}} + \phi _{ls}^{par} \, [{\mathbb{I}} \; + \\&{\mathbb{P}}_{sphrd}^{par,(1)}:({{\mathbb{c}}}_w - {\mathbb{C}}_{par}^{(1)})]^{-1} \Big \}^{-1}. \end{aligned} \end{aligned}$$

#### Sclerenchyma fibers and vascular bundles

In the RVE of the sclerenchyma fibers (level $b_2$ in Fig. 5), we denote the volume fractions of the cell wall material in the matrix phase as $\phi _{wall}^{fib}$ and the volume fraction of the lumen inclusions as $\phi _{lum}^{fib}$, where $\phi _{wall}^{fib} + \phi _{lum}^{fib} = 1$. The stiffness of the lumen material is the same as stiffness ${{\mathbb{c}}}_w$ of water. The RVE can be suitably modeled by the Mori-Tanaka scheme. Hence, we assume that ${\mathbb{C}}^0 = {\mathbb{C}}_{wall}^{fib}$ and ${\mathbb{P}}_r^0 = {\mathbb{P}}_{cyl}^{wall}$, which corresponds to cylindrical inclusions in the transversely isotropic matrix of the cell wall material in sclerenchyma fibers (Fritsch et al. 2009). Following (12), the stiffness tensor ${\mathbb{C}}_{fib}^{}$ for the RVE of the sclerenchyma region can be obtained as

25 $$\begin{aligned} \begin{aligned} {\mathbb{C}}_{fib}^{}= \Big \{ & \phi _{wall}^{fib} {\mathbb{C}}_{wall}^{fib} + \phi _{lum}^{fib} {{\mathbb{c}}}_w:[{\mathbb{I}} + {\mathbb{P}}_{cyl}^{wall}: \\& ({{\mathbb{c}}}_w - {\mathbb{C}}_{wall}^{fib})]^{-1} \Big \} : \Big \{ \phi _{wall}^{fib} {\mathbb{I}} + \phi _{lum}^{fib} \, [{\mathbb{I}} \; + \\&{\mathbb{P}}_{cyl}^{wall}:({{\mathbb{c}}}_w - {\mathbb{C}}_{wall}^{fib})]^{-1} \Big \}^{-1}. \end{aligned} \end{aligned}$$

In the RVE of the *vascular bundle* (level *c* in Fig. 5), we denote the volume fractions of the sclerenchyma fibers and the vessels as $\phi _{fib}^{}$ and $\phi _{v}^{}$, where $\phi _{fib}^{} + \phi _{v}^{} = 1$. The vessels form cylindrical inclusions in the matrix of sclerenchyma fibers, which motivates the use of the Mori-Tanaka scheme. We are given the Hill tensor ${\mathbb{P}}_r^0 = {\mathbb{P}}_{cyl}^{fib}$, which corresponds to cylindrical inclusions in the transversely isotropic matrix of the sclerenchyma fibers (Fritsch et al. 2009). Hence, with ${\mathbb{C}}^0 = {\mathbb{C}}_{fib}^{}$, the elastic stiffness tensor ${\mathbb{C}}_{vb}^{}$ of a vascular bundle RVE can be determined as

26 $$\begin{aligned} \begin{aligned} {\mathbb{C}}_{vb}^{}&= \Big \{ \phi _{fib}^{} {\mathbb{C}}_{fib}^{} + \phi _{v}^{} {{\mathbb{c}}}_w:[{\mathbb{I}} + {\mathbb{P}}_{cyl}^{fib}: ({{\mathbb{c}}}_w - {\mathbb{C}}_{fib}^{})]^{-1} \Big \} : \\&\quad \Big \{ \phi _{fib}^{} {\mathbb{I}} + \phi _{v}^{} [{\mathbb{I}} + {\mathbb{P}}_{cyl}^{fib}:({{\mathbb{c}}}_w - {\mathbb{C}}_{fib}^{})]^{-1} \Big \}^{-1}. \end{aligned} \end{aligned}$$

#### Soft-pith and outer-shell materials

The macroscopic section of the crop stem material is made up of a soft-pith core surrounded by outer-shell material (see Fig. 5c). In the RVE of the soft-pith region (level *d* in Fig. 5), the vascular bundles are embedded into the matrix of parenchyma base tissues. The volume fraction of the parenchyma region and the vascular bundles are $\phi _{par}^{}$ and $\phi _{vb}^{}$, such that $\phi _{par}^{} + \phi _{vb}^{} = 1$. The RVE can be suitably modeled by the Mori-Tanaka scheme. Hence, we assume that ${\mathbb{C}}^0 = {\mathbb{C}}_{par}^{}$ and the Hill tensor ${\mathbb{P}}_r^0 = {\mathbb{P}}_{cyl}^{par}$, which corresponds to cylindrical inclusions in the transversely isotropic matrix of the parenchyma (Fritsch et al. 2009). Following (12), we arrive at the homogenized stiffness tensor ${\mathbb{C}}_{pith}$ of the soft-pith as

27 $$\begin{aligned} \begin{aligned} {\mathbb{C}}_{pith}^{}= \Big \{&\phi _{par}^{} {\mathbb{C}}_{par}^{} + \phi _{vb}^{} {\mathbb{C}}_{vb}:[{\mathbb{I}} + {\mathbb{P}}_{cyl}^{par}: \\& ({\mathbb{C}}_{vb} - {\mathbb{C}}_{par}^{})]^{-1} \Big \} : \Big \{ \phi _{par}^{} {\mathbb{I}} + \phi _{vb}^{} [{\mathbb{I}} \; + {\mathbb{P}}_{cyl}^{par}:\\&({\mathbb{C}}_{vb} - {\mathbb{C}}_{par}^{})]^{-1} \Big \}^{-1}. \end{aligned} \end{aligned}$$

Figure 5c shows a multistep model for the outer-shell material. The RVE of the cell wall material in this model (level *e* in Fig. 5) is identical to the RVE level $(a_2)$. In the RVE of the *outer-shell* at level (*f*) , we denote the volume fractions of the cell wall material and lumen as $\phi _{wall}^{shell}$ and $\phi _{lum}^{shell}$, where $\phi _{wall}^{shell} + \phi _{lum}^{shell} = 1$. The RVE can be modeled by the Mori-Tanaka scheme. The stiffness of the lumen material is the same as the stiffness ${{\mathbb{c}}}_w$ of water. With ${\mathbb{C}}^0 = {\mathbb{C}}_{wall}^{fib}$ from (22) and ${\mathbb{P}}_r^0 = {\mathbb{P}}_{cyl}^{wall}$ and following (12), the stiffness tensor for the RVE of the outer shell is

28 $$\begin{aligned} \begin{aligned} {\mathbb{C}}_{shell}^{} =&\Big \{ \phi _{wall}^{shell} {\mathbb{C}}_{wall}^{fib} + \phi _{lum}^{shell} {{\mathbb{c}}}_w:[{\mathbb{I}} + {\mathbb{P}}_{cyl}^{wall}: \\&({{\mathbb{c}}}_w - {\mathbb{C}}_{wall}^{fib})]^{-1} \Big \} : \Big \{ \phi _{wall}^{shell} {\mathbb{I}} + \phi _{lum}^{shell} [{\mathbb{I}} + {\mathbb{P}}_{cyl}^{wall}: \\&({{\mathbb{c}}}_w - {\mathbb{C}}_{wall}^{fib})]^{-1} \Big \}^{-1}. \end{aligned} \end{aligned}$$

### Upscaling elastic limit strength in crop stem material

In the next step, we estimate the elastic limit strength of the soft-pith and outer-shell materials in the crop stem cross-section, following the discussion in Sect. 3.1.2. The elastic limit point in wood and grass stems correspond to the yielding of lignin at microscales (Hofstetter et al. 2008; Gangwar and Schillinger 2019). Therefore, we assume that the soft-pith material and the outer-shell material reach the limit state when the elastic limit is reached in the lignin phase at the microscales. Lignin is an amorphous material, and it is known to fail in shear (Bader et al. 2010). The shear strength of lignin $s_{lig}$ is reported as 20.2 MPa (Bader et al. 2010). Its stress–strain response is assumed to be first elastic and then perfectly plastic, following the von Mises failure criterion expressed as

29 $$\begin{aligned} \varvec{{\mathfrak {f}}}_{lig}(\varvec{\sigma }_l) = \sigma ^2_{eq(l)} - 3s_{lig}^2 \le 0, \end{aligned}$$

$\sigma _{eq(l)}$ is the von Mises equivalent stress of the quadratic stress average in the lignin phase, which can be evaluated from (17).

#### Elastic limit of the soft-pith material

Algorithm 1 outlines how to compute the elastic limit strength of the soft-pith material. The parenchyma base material and the vascular bundles come together to form the soft-pith material (level *d* in Fig. 5). Both parenchyma and vascular bundle branches have lignin at the lowermost cell wall material levels. Therefore, the yielding of lignin within any one of parenchyma or vascular bundle tissues determines the elastic limit state of the soft-pith material. 
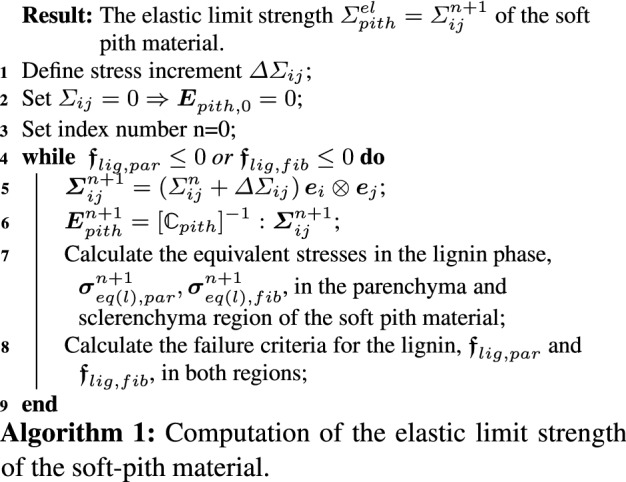


By analogy to (18), we write the von Mises equivalent of the quadratic stress averages $\sigma _{eq(l),par}$ in the lignin phase embedded in the parenchyma tissues as

30 $$\begin{aligned} \frac{\sigma _{eq(l),par}}{\mu _{lig}} = \left( \frac{1}{{\overline{\phi }}_{lig,par}} {\varvec{E}}_{pith}: \frac{\partial \; {\mathbb{C}}_{pith}}{\partial \mu _{lig,par}}:{\varvec{E}}_{pith}\right) ^{1/2}. \end{aligned}$$

where ${\overline{\phi }}_{lig,par}$ is the equivalent volume fraction of the lignin in the parenchyma, computed as ${\overline{\phi }}_{lig,par} = \phi _{par} \cdot \phi ^{par}_{wall} \cdot \phi ^{wall,par}_l$. The derivative term $\partial {\mathbb{C}}_{pith}/\partial \mu _{lig,par}$ denotes the change in the elasticity tensor of soft-pith material with respect to the change in the shear modulus of the lignin phase in the cell wall material of the parenchyma region. This derivative can be evaluated via a finite difference approximation (see Appendix 3 in Pichler et al. (2008)). Uniform macrostrain ${\varvec{E}}_{pith}$ imposed on the RVE of the soft-pith material is related to the macrostress $\varvec{{\varSigma }}_{pith}$ as

31 $$\begin{aligned} {\varvec{E}}_{pith} = [{\mathbb{C}}_{pith}]^{-1}:\varvec{{\varSigma }}_{pith} . \end{aligned}$$

We emphasize that we do not “successively” propagate from one RVE to the other. The term $\partial {\mathbb{C}}_{pith}/\partial \mu _{lig,par}$ implicitly accounts for all hierarchical scales and directly provides access down to the lignin phase. In Pichler and Hellmich (2011), a similar approach is used for upscaling the strength of cement paste and mortar. The failure criterion for the lignin in the parenchyma region follows from (29) as

32 $$\begin{aligned} \varvec{{\mathfrak {f}}}_{lig,par} = \sigma ^2_{eq(l),par} - 3s_{lig}^2 \le 0 . \end{aligned}$$

Similarly, we write the von Mises equivalent of the quadratic stress average $\sigma _{eq(l),fib}$ in the lignin phase in the fibers embedded in the bundles as

33 $$\begin{aligned} \frac{\sigma _{eq(l),fib}}{\mu _{lig}} = \left( \frac{1}{{\overline{\phi }}_{lig,fib}} {\varvec{E}}_{pith}: \frac{\partial \; {\mathbb{C}}_{pith}}{\partial \mu _{lig,fib}}:{\varvec{E}}_{pith}\right) ^{1/2}. \end{aligned}$$

where ${\overline{\phi }}_{lig,fib}$ is the equivalent volume fraction of the lignin in the bundle regions, computed as ${\overline{\phi }}_{lig,fib} = \phi _{vb} \cdot \phi _{fib} \cdot \phi ^{fib}_{wall} \cdot \phi ^{wall,fib}_l$. The corresponding failure criterion is

34 $$\begin{aligned} \varvec{{\mathfrak {f}}}_{lig,fib} = \sigma ^2_{eq(l),fib} - 3s_{lig}^2 \le 0. \end{aligned}$$

The stress level $\varvec{{\varSigma }}_{pith}$ that violates (31) or (33) is the elastic limit strength ${\varSigma }^{el}_{pith}$ of the soft-pith region (see also Algorithm 1).

#### The elastic limit of the outer-shell region

Following the above discussion, determining the elastic limit strength of the outer shell (see Fig. 5) is straightforward. We can write the von Mises equivalent of the quadratic stress average $\sigma _{eq(l),shell}$ in the lignin phase as a function of the macroscopic strain ${\varvec{E}}_{shell}$ imposed on the RVE as

35 $$\begin{aligned} \frac{\sigma _{eq(l),shell}}{\mu _{lig}} = \left( \frac{1}{{\overline{\phi }}_{lig,shell}} {\varvec{E}}_{shell}: \frac{\partial \; {\mathbb{C}}_{shell}}{\partial \mu _{lig,shell}}:{\varvec{E}}_{shell}\right) ^{1/2} \end{aligned}$$

where ${\overline{\phi }}_{lig,shell}$ is the equivalent volume fraction of lignin in the outer-shell region, computed as ${\overline{\phi }}_{lig,shell} = \phi ^{shell}_{wall} \cdot \phi ^{wall,fib}_l$. The macroscopic strain ${\varvec{E}}_{shell}$ can be expressed in terms of macrostress $\varvec{{\varSigma }}_{shell}$ with the help of the homogenized elasticity tensor ${\mathbb{C}}_{shell}$ as

36 $$\begin{aligned} {{\varvec{E}}}_{shell} = [{\mathbb{C}}_{shell}]^{-1}:\varvec{{\varSigma }}_{shell}. \end{aligned}$$

Corresponding failure criterion $\varvec{{\mathfrak {f}}}_{lig,shell}$ reads as:

37 $$\begin{aligned} \varvec{{\mathfrak {f}}}_{lig,shell} = \sigma ^2_{eq(l),shell} - 3s_{lig}^2 \le 0. \end{aligned}$$

Equation (37) together with (35) and (36) affects the macroscopic failure criterion for the outer-shell region. The stress level $\varvec{{\varSigma }}_{shell}$ that results in the failure of lignin according to (37) is the elastic limit strength $\varvec{{\varSigma }}^{el}_{shell}$ of the outer-shell region.

## Results and discussion

In this section, we first validate our micromechanics-based model against four-point bending tests that we performed on oat stems. To this end, we integrate our multiscale material model with macroscale finite element analysis. We then illustrate the potential of our model for simulating and understanding the biomechanical tailoring of crop stems. To this end, we quantify the effect of genetic modifications on the mechanical properties of thale cress as predicted by our micromechanics model and compare the predictions with experimental evidence reported in the literature.

**Fig. 6:**
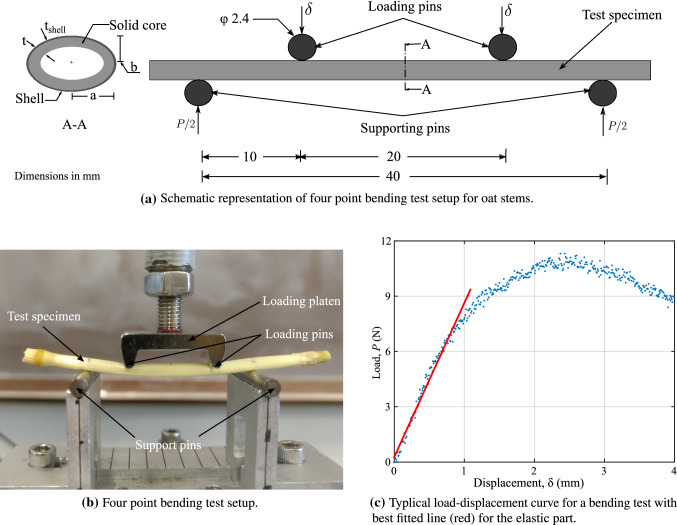
Material characterization of oat stems

**Fig. 7:**
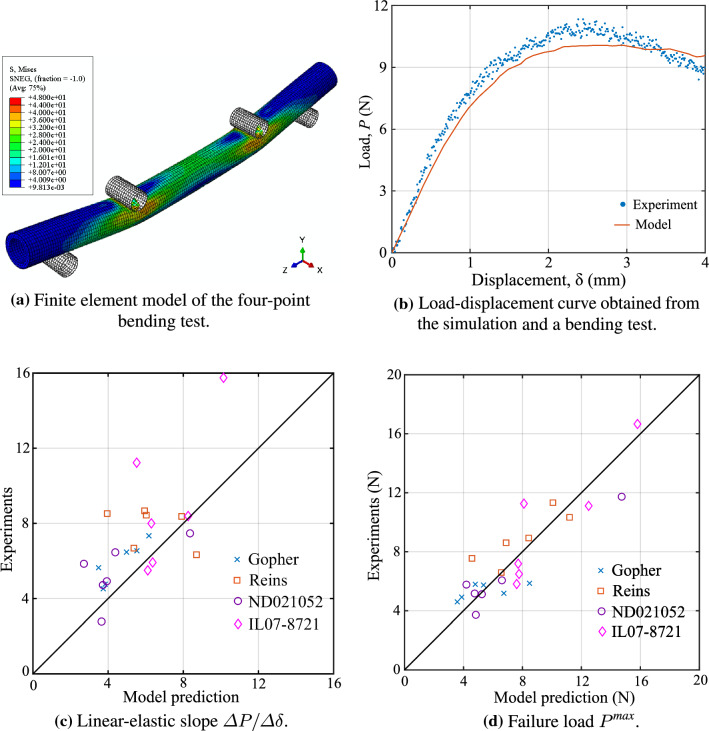
Comparison of results from the micromechanics model and the experimental tests

### Model validation against four-point bending experiments with oat stems

To validate our micromechanics model against crop stem experiments, we conducted a series of standard four-point bending tests with oat stems. The stem specimens used in the tests were grown in the fields at the University of Minnesota in St. Paul, MN. Four commercial varieties of oats—Gopher, Reins, ND021052, and IL07-8721—were planted in the summer of 2017 with four different sowing dates for each variety. Plants were randomly selected for the tests including all of the varieties. The plants were carefully plucked with visually intact roots and kept for 24 h with the roots submerged in water. Test specimens were prepared from different internode locations for each plant. The leaf sheath was carefully removed, and adjacent nodes were kept to maintain the integrity of the specimens. Only specimens with no visible damage were included in the four-point bending tests.

Figure 6a shows the schematic diagram of the four-point bending test apparatus. The total span length and loading span length were fixed at 40 mm and 20 mm. The oat stem specimen can be approximated as a hollow cylinder with an elliptical cross-section. The major axis was kept perpendicular to the transverse loading axis in the tests. The major axis a, the minor axis b, and the thickness t were measured at different locations with Vernier calipers, and the average values are reported in B. The tests were conducted via a universal testing frame (MTS Instron 858 Mini Bionix II) with a 500 N load cell. The loading platen was displaced at a slow rate of 0.01 mm/s during the tests (see Fig. 6b). Load P and displacement data were recorded every second until a clear failure of the specimen. Failure is when the specimen loses structural integrity after a complete ovalization of the cross-section under the loading pins. Thereafter, the load begins to decrease with an increasing deflection in the recorded load–displacement curve (Fig. 6c). The failure load along with the slope of the load–displacement curve in the linear elastic region (red line in Fig. 6c) is reported in B.

We then build a finite element model for each specimen in the commercial software ABAQUS, discretizing the inner solid core region with twenty-node brick elements (C3D20R) and the outer-shell region with eight-node shell elements (S8R), respectively. Since the basis functions of the shell conform with those of the solids at the coupling surface, surface locking between shell and solid elements is prevented (Schillinger et al. 2018). Once the model parameters are known for each oat variety (see B), we calculate and assign the macroscopic elastic stiffness moduli and strength properties for both the solid core and shell region. We model the loading and supporting pins using three-dimensional rigid elements (R3D4). We define surface-to-surface interactions between the specimen model and the rigid pins with a tangential friction coefficient of 0.2. We fix the support pins and apply the vertical displacement at the loading pins, keeping other displacement components zero. We perform nonlinear finite element analysis using the displacement control static-general algorithm in ABAQUS. We also considered the nonlinear geometric effects in the simulations. A typical load–displacement curve for one of the specimens obtained from the simulation and from the experimental observations is shown in Fig. 7b. A linear part, a peak and a softening branch are apparent in this figure. The softening branch is a result of the interaction of geometric nonlinearities and material yielding. However, in this work, we mainly focus on the linear elastic slope and peak failure load predictions. In the softening region, large macroscopic deformation gradients may invoke second-order effects on the microscales. In the scope of the present work, we do not account for these effects in the material model.

Figure 7c, d plots 24 four-point bending simulation predictions versus the experimental values for the linear elastic slope and the failure load, respectively. These plots show good agreement between the simulations and the experiments. The correlation coefficient *R* between the experimental and simulation results for the linear-elastic slope and failure load is 0.65 and 0.88, respectively. We also compute the mean percentage difference and standard deviation of the relative difference in percent between the experimental results and model predictions,

38 $$\begin{aligned} {\bar{e}}&= \frac{1}{n}\sum e_i = \frac{1}{n}\sum \frac{q_i^{exp} - q_i^{pred}}{q_i^{exp}} \times 100 , \end{aligned}$$

39 $$\begin{aligned} e_{sd}&= \left[ \frac{1}{n-1}\sum { (e_i - {\bar{e}} )^2}\right] ^{1/2} , \end{aligned}$$

where q is either the linear elastic slope or the failure load. We find that the relative difference ${\bar{e}}\pm e_{sd}$ is in the range of $16.9 \pm 23.9\%$ for the linear elastic slope and in the range $-0.4 \pm 22.0\%$ for the failure load. The variance in the experimental observations and model predictions is mainly because of possible measurement and experimental errors. Overall, the results confirm that our material model is able to accurately predict the stiffness and strength properties of oat stem sections.

**Fig. 8:**
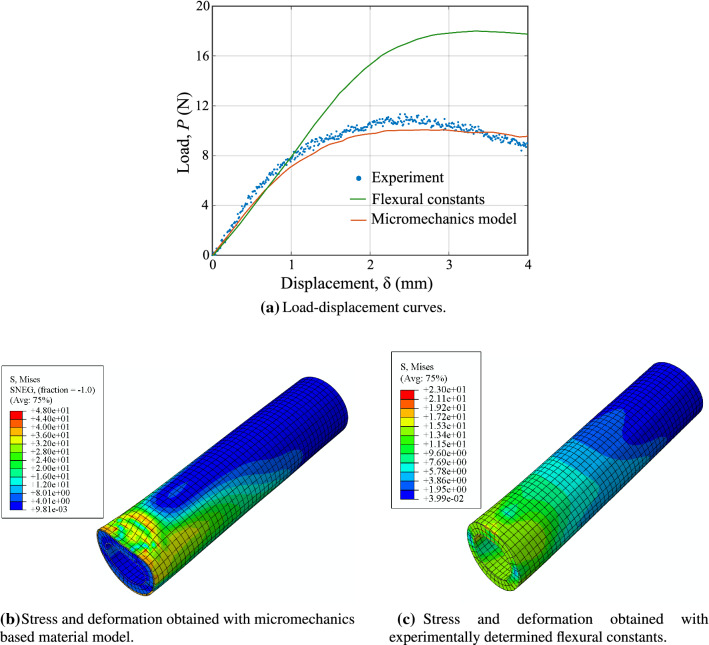
Comparison of simulation results based on our micromechanics material model and based on experimentally calibrated flexural constants

### Model comparison with isotropic flexural constants

To emphasize the importance of a properly calibrated multiscale material model, we compare the model predictions obtained with our micromechanics based model against predictions based on standard isotropic material constants. Using standard beam flexural theory and the available experimental results, one can compute Young’s modulus to be $L^3 ( {\varDelta }P/ {\varDelta }\delta ) / (96\, I)$, where ${\varDelta }P/ {\varDelta }\delta$ is the linear-elastic slope of the load–displacement curve, *L* is the span length, and *I* is the second moment of area of the section. The elastic limit strength of the material follows from $P^{\textit{max}}Lb/4I$, where $P^{\textit{max}}$ is the maximum force value in the load–displacement curve. We can now use these material parameters in the finite element simulations, where we obtain the load–displacement curve shown in Fig. 8a. We observe, however, that the simulated material response based on flexural theory based isotropic constants grossly overestimates the maximum strength.

The reason for the large deviation can be traced back to the assumption of isotropy, where the two elastic material parameters are associated with the axial response of the structure. Taking the same constants in all directions, the structural response in the transverse direction, which is essential to resist ovalization and thus local buckling, is stiffer than it is in reality. The consequence is illustrated via the deformation and stress plots in Fig. 8b, c that show a quarter of a simulated structure from one of the supports to the nearest leading pin location. While the result based on our micromechanics-based material model clearly indicates ovalization, the result based on the experimentally calibrated material model still maintains an intact circular cross-section. Therefore, taking into account the anisotropy and associated material parameters is essential to accurately predict the mechanical response of plant stems. Anisotropy in plant materials is the outcome of different microstructures across several scales. Our micromechanics-based model naturally accounts for the anisotropic material properties from the underlying plant physiology, accurately predicting their effects on the macroscale mechanical behavior.

### Predicting and explaining the mechanical behavior of crop mutants

The stem material properties depend on the specific combination of morphological and compositional parameters that vary across different length scales from the cell wall to the cross-section level. Brule and co-authors (Brulé et al. 2016) reviewed the effect of genetic modification of these parameters on the stiffness and strength for the genetic reference plant *Arabidopsis thaliana* (Arabidopsis), commonly known as thale cress. In their study, a consistent explanation of their findings was not possible as the genetic modifications affected the plant structure and the physiological response at multiple levels. The authors concluded: “*What is needed is a comprehensive, systematic and consistent multiscale mechanical analysis of structural parameters across length scales to feed into an integrated model of the development of plant stiffness.*”

In Table 3, we compile experimentally determined changes in the mechanical properties of the primary cell wall, secondary cell wall, and functional region mutants of *Arabidopsis* from Brulé et al. (2016). In the following, we demonstrate that our multiscale modeling approach is capable of rationally interpreting and quantifying the effect of compositional and morphological parameters across multiple length scales on the mechanical response of *Arabidopsis*.

#### Remark 1

We note that for the *Arabidopsis* mutant tensile test samples, not all of the required parameters are available. To enable model predictions, we chose our base specimen as the oat variety Gopher with measured parameters reported in Appendix B. We characterize morphological and compositional changes in the respective mutants and assume that oat will experience similar changes in the composition and morphology against similar genetic alterations. We calculate and apply these changes in our model to predict the axial stiffness and the strength of the “mutant” oat. In Table 3, we report percentage changes in the stiffness and strength compared to our base specimen and use these results in the following discussion.

**Table 3:** Comparison of model predictions with experiments for crop mutants

Gene	Phenotype	Experiment (% of WT)		Model (% of Base)		Reference
		Stiffness	Strength	Stiffness	Strength	
*Primary cell wall (expressed in parenchyma cell wall) mutants:*						
*atkin/frac2/bot*	Reduced cellulose	40–60%	40–60%	66%	71%	Ryden et al. (2003)
*mur2/xxt1xxt2*	Reduced hemicellulose	80–100%	80%	85%	85%	Ryden et al. (2003), Burgert and Dunlop (2011), Cavalier et al. (2008)
*qua2*	Reduced pectin	80–100%	100%	NA	NA	Burgert and Dunlop (2011), Abasolo et al. (2009)
*Second cell wall (expressed in fiber cell wall) mutants:*						
*cesa7/irx3/frac5*	Reduced cellulose	20–40%	60–80%	56%	58%	Turner and Somerville (1997)
*irx4/ccr1*	Reduced lignin	40%	40%	56%	65%	Jones et al. (2001)
*Functional region (vascular bundles, sclerenchyma fibers, outer-shell tissues) mutants:*						
*parvus/gatl1/irx7/frac8*	Reduced xylan	NA	20%	40%	40%	Zhong et al. (2005), Peña et al. (2007), Lee et al. (2007)
*abv1/ifl1/rev*	Modified bundle arrangements	NA	60%	68%	70%	Zhong and Ye (2004)
*ifl1/rev*	Missing outer-shell	NA	20%	48%	48%	Zhong et al. (1997)

#### Primary cell wall mutants

Experimental tests in Table 3 indicate a reduction in the stiffness and strength as a result of mutations that reduce constituent materials in the primary cell wall. For experimental determination, one can use tensile tests on hypocotyl tissues (Ryden et al. 2003). The tissues were obtained from the basal region of *Arabidopsis* in the early growth stage with the assumption that no secondary cell wall growth has happened. Cell wall material in the parenchyma region is largely made up of primary cell walls. Therefore, the parenchyma region from our micromechanics model that consists of the RVEs $(a_1)$ and $(b_1)$ shown in Fig. 5 can represent the test specimen. We use (20) and (24) to derive stiffness tensor ${\mathbb{C}}_{par}$ and the axial stiffness modulus as $1/{\mathbb{C}}^{-1}_{par,33}$, where ${\mathbb{C}}^{}_{par,33}$ is the longitudinal component of the stiffness tensor ${\mathbb{C}}_{par}$. We estimate the axial strength following the procedure discussed in Sect. 3.4 and Algorithm 1.

A reduction in cellulose content results in a drastic decrease in stiffness and strength. In Ryden et al. (2003), a 40% decrease in the cellulose content was reported due to the presence of a cellulose synthesis inhibitor gene. This alteration resulted in a 40% axial stiffness and strength decrease with respect to the respective wild-type (WT) specimen. We can now use our model to predict the new cell wall fraction parameters in (20), i.e., $\phi ^{wall,par}_{i}$ with $i \in [cc,h,l]$, and $\phi ^{par}_{wall}$ in (24). The axial stiffness and strength are computed with the parameters that correspond to mutants with 40% reduced cellulose. The stiffness and strength values are reduced to 66% and 71% compared to the base oat material. In Table 3, we observe that the model predictions are in line with the experimental results. Our observations reconfirm the role of cellulose as a load-bearing polymer in the cell wall.

Mutants *mur2* and *xx1xx2* show a significant reduction in the hemicellulose component xyloglucan in the primary cell wall (Ryden et al. 2003; Burgert and Dunlop 2011). The corresponding loss of stiffness and strength, however, is not as severe as in the case of reduced cellulose (see Table 3). To enable a comparison with cellulose reduction, we assume an identical 40% reduction in hemicellulose content in the cell wall material. Using our model, we find that the axial stiffness and strength is reduced to 85% of the base oat material, consistent with the experimental observations. This result is plausible as the axial stiffness modulus of hemicellulose is approximately 15 times smaller than that of cellulose. Therefore, cellulose contributes to a much larger extent to the macroscale mechanical properties than hemicellulose. We anticipate that our micromechanics model could help settle the current controversial discussion in the literature about the role of hemicellulose as a load-carrying polymer in the cell wall material (Park and Cosgrove 2015; Cosgrove 2015).

Pectin, a major component of middle lamella, is a complex set of polysaccharides. Pectin-rich middle lamella forms a continuous layer between the adjacent cells in the primary cell wall (see Fig. 4b). This layer presumably acts as a binding agent between the cells (Braidwood et al. 2014). Table 3 indicates a minimal effect on the stiffness and strength in the reduced pectin mutants. Our model does not explicitly account for the role of middle lamella in the homogenized properties. A fundamental assumption of the continuum micromechanics frameworks, however, is perfect bond between the different phases in a RVE. The experimental results in Table 3 suggest that if “sufficient” pectin is present in the middle lamella to ensure perfect bond, it will not have a major effect on the overall stiffness and strength properties. For a detailed quantitative investigation on the role of pectin, however, more detailed computational models supported by physiological investigations at relevant length scales are needed.

#### Secondary cell wall mutants

Secondary cell wall mutants result in a loss of stiffness and strength as shown in Table 3. For mechanical testing, one can use three-point bending tests (Turner and Somerville 1997; Jones et al. 2001). The reported strength corresponds to $P^{\textit{max}}Lr/4I$, where $P^{\textit{max}}$ is the maximum force the sample withstands before failure, *L* is the span length, *r* is the radius of the stem, and *I* is the second moment of area. The axial stiffness modulus is simply $L^3 ( {\varDelta }P/ {\varDelta }\delta ) / (48I)$, where ${\varDelta }P/ {\varDelta }\delta$ is the linear-elastic slope of the load–displacement curve. For consistent comparison, we simulate the three-point bending test, where we follow the procedure outlined in Sect. 4.2, using the whole-stem specimen. The complete model described in Sect. 3 is used to predict the material parameters for the mutants after accommodating the compositional and morphological changes. Based on our simulations results, we report percentage changes in stiffness and strength compared to the oat base specimen.

Cellulose is a major load-bearing polymer in the secondary cell walls. A reduction in the cellulose content is expected to decrease stiffness and strength, as shown in Table 3. In Turner and Somerville (1997), the percentage of cellulose content in the cell wall is reported as low as 18% of the WT variety after genetic alteration. The reduction is also reflected in the thinning of the secondary cell walls in the fibers (Figs. 3 and 6 in Turner and Somerville 1997). Using our model, we recalculate the volume fractions in the RVEs that involve secondary cell walls, considering the mutant cellulose content (18% of the WT variety). The resulting stiffness and strength parameters are fed in the three-point bending simulations and the percentage change is reported in Table 3. We observe that the model predictions are in good agreement with the experimental observations, confirming the importance of cellulose for the overall stiffness and strength of plants.

Lignin reduction is a crucial process in various bioengineering applications such as biomass conversion to paper, fuel, and cattle feedstock. The genetic reduction in lignin content, however, results in a drastic loss of stiffness and strength as summarized in Table 3. In Jones et al. (2001), a 50% reduction in lignin content in the secondary cell wall is reported as a consequence of mutant gene *irx4*. In addition, other morphological changes were observed such as the reduction of sclerenchyma fiber fractions in bundles and physiological changes in the outer-shell region (Figs. 2 and 5 in Jones et al. 2001). To predict and quantify the effect of these changes, we use the three-point bending simulation, where we assume a 50% reduction in fiber volume fraction $\phi _{fib}$ in the bundles and shell thickness $t_{shell}$. Based on these changes, we predict a 56% and 65% reduction in stiffness and strength, respectively, compared to the base oat material. These results confirm the importance of considering morphological changes at multiple levels in mutants. In Horvath et al. (2010), Koehler and Telewski (2006); Köhler and Spatz (2002), similar observations in reduced lignin mutants for wood are reported.

#### Functional region mutants

Using our model, we finally assess the stiffness and strength impact of mutants that result in morphological changes in the functional regions such as vascular bundles, sclerenchyma fibers, and outer-shell tissues. Table 3 indicates a severe reduction in the axial strength as a consequence of these mutants. We note that the axial strength was measured through tensile tests with matured whole-stem specimens. In each case, we accommodate the observed changes in the morphology for the specific mutants in the model and predict the stiffness and strength for both pith and outer-shell region. For comparison, we compute the volumetric average of the predicted axial stiffness and strength components of the pith and outer-shell properties.

Xylans are found in sclerenchyma fibers in bundles and outer-shell tissues. The disruption of genes involved in xylan synthesis leads to a reduction of up to 50% of the WT xylan level and results in a severe decrease in tensile strength (see Table 3). Xylan mutants such as *PARVUS* resulted in compositional and morphological changes in the hierarchical structure of the reference plant (Lee et al. 2007). A reduction of 50% and 64% cell wall thickness was reported in the sclerenchyma fibers in the bundles and outer-shell regions, respectively. Overall, a 25% reduction in the cellulose content in the cell walls was reported. We incorporate these changes in our model and modify $\phi ^{fib}_{wall}$, $\phi ^{shell}_{wall}$, and the constituent cell wall fractions, i.e., $\phi ^{wall,fib}_{cc}$ with $i \in [cc,h,l]$ (see Appendix B for details), leading to a 60% reduction in the axial stiffness and strength. These results confirm the drastic changes in the mechanical properties as a result of xylan mutants.

The spatial arrangement and proportion of tissue types in the cross-section of crop stems affect their mechanical properties. The mutant *abv1* (Zhong and Ye 2004) alters the organization of vascular bundles from a “collateral pattern” to an “amphivasal pattern.” In the collateral pattern, bundles are arranged in a ring-like configuration near the periphery of the cross-section. In the amphivasal pattern, bundles are irregularly distributed in the pith. As a result of this mutation, a gross thinning of the outer-shell fiber cell wall was also observed (see Fig. 6 in Zhong and Ye 2004). Assuming a 60% reduction in the cell wall fraction in outer-shell tissues, our model predicts a 30% reduction in the axial stiffness and strength of the stem cross-section. Similarly, a diminished outer shell is reported in Zhong et al. (1997) after *ifl1/rev* mutation, resulting in the severe loss of axial strength as reported in Table 3. Our model predicts the axial stiffness and strength as 48% of the base oat material for this alteration in the cross-section morphology. This result is consistent with experimental observations.

## Summary, conclusions and outlook

In this article, we presented a multiscale material model that predicts the stiffness and strength of crop stem materials directly from the interaction of hierarchical microstructures and associated parameters. Building on our prior work on bamboo that focused on functionally graded type materials, we focused here on the configuration typical for crop stems that consists of an inner foam-like parenchyma layer and a dense outer shell. We first reviewed the hierarchical organization of crop stem materials for the prototypical example of oats, and derived a set of morphological and material data at different length scales with the help of chemical analysis and imaging data from transmission electron microscopy, light microscopy, and micro-CT scans. We then transferred this data into representative micromechanical parameters at different length scales (e.g., volume fractions, shape, orientation, distribution, elastic and failure properties of constituents, etc.). We used this set of parameters at different scales to motivate a sequence of RVEs that enables the prediction of the stiffness and elastic limit properties of crop stem materials within the framework of continuum micromechanics. We validated our model against bending experiments that we conducted for oat stem samples grown in a controlled environment on test fields at the University of Minnesota. The predictions from our multiscale material model built in the ABAQUS finite element solver showed very good agreement with the experimental results, both with respect to the stiffness in the linear elastic range and the failure load in the inelastic range.

A significant advantage of our multiscale model over simpler phenomenological approaches is the physiologically correct relation of the mechanical properties with compositional and morphological parameters across multiple length scales. This connection enables an understanding of the multiscale origin of stiffness and strength. We demonstrated this opportunity by explaining the effect of genetic modifications on the stiffness and strength reported for thale cress from our model that predicted the observed behavior correctly in all cases. From a plant breeding perspective, this connection opens the door for tailoring plant materials in situations where their mechanical properties are of key importance. In the future, we plan to use our micromechanics model to help identify optimal material and geometric traits that maximize lodging resistance of cereals.

## Figures and Tables

**Fig. 9:**
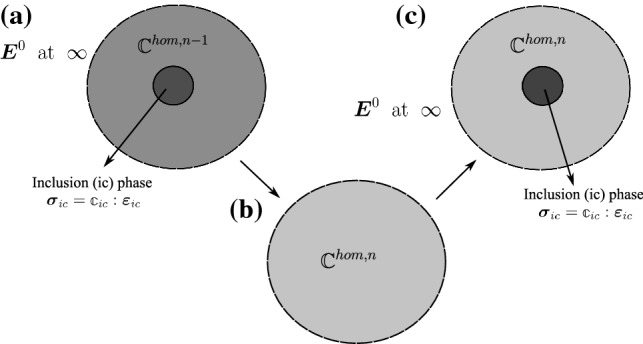
Outline of the cascade continuum micromechanics scheme: **a** matrix-inclusion problem with matrix stiffness ${\mathbb{C}}^{hom,n-1}$, **b** homogenized composite with stiffness ${\mathbb{C}}^{hom,n}$, **c** matrix-inclusion problem with updated matrix stiffness ${\mathbb{C}}^{hom,n}$ obtained from step (**b**) (for more details see Timothy and Meschke 2016)

## Appendices

### A. Cascade continuum micromechanics model

In the cascade continuum micromechanics (CCM) model (see Fig. 9), a set of matrix-inclusion problems are obtained through recursion Timothy and Meschke (2016, 2017). The CCM model is inspired by the self-consistent scheme. Instead of the virtual “average” matrix phase, the stiffness of the matrix phase at cascade level *n* is recursively updated and set equal to the previously homogenized stiffness (see Fig. 9). At all cascade levels, inclusion properties are assumed to remain the same.

We assume that there are two phases in a RVE, namely inclusion and matrix, with volume fractions $\phi _{ic}$ and $\phi _{m}$, respectively, and $\phi _{ic} + \phi _{m} = 1$. We denote inclusion and matrix stiffness properties with ${{\mathbb{c}}}_{ic}$ and ${{\mathbb{c}}}_{m}$, respectively. Following (11) and (12), we can write the homogenized stiffness ${\mathbb{C}}^{hom,(n)}$ at cascade level *n* after setting the matrix stiffness equal to the homogenized stiffness obtained from the previous homogenization step (${\mathbb{C}}^0 = {{\mathbb{c}}}_{m} = {\mathbb{C}}^{hom,(n-1)}$) as

40 $$\begin{aligned} \begin{aligned} {\mathbb{C}}^{hom,(n)} =&\Big [\phi _m \; {\mathbb{C}}^{hom,(n-1)} + \phi _{ic} \; {{\mathbb{c}}}_{ic}:[{\mathbb{I}} + {\mathbb{P}}_{ic}^{hom,(n-1)}: \\&({{\mathbb{c}}}_{ic} - {\mathbb{C}}^{hom,(n-1)})]^{-1}\Big ]: \Big [\phi _{m}\; {\mathbb{I}} + \phi _{ic} \; [{\mathbb{I}} \, + \\& {\mathbb{P}}_{ic}^{hom,(n-1)}:({{\mathbb{c}}}_{ic} -{\mathbb{C}}^{hom,(n-1)})]^{-1} \Big ]^{-1}. \end{aligned} \end{aligned}$$

The Hill tensor ${\mathbb{P}}_{ic}^{hom,(n-1)}$ describes the morphological description of the inclusion in the matrix with elastic stiffness ${\mathbb{C}}^{hom,(n-1)}$.

Equation (40) represents the recursive equation for the homogenized stiffness of the RVE. The initial configuration at $n = 1$ is assumed to be a continuous matrix with a disconnected distribution of the inclusions. The homogenized elastic properties for $n = 1$ is provided by the Mori-Tanaka scheme (${\mathbb{C}}^{hom,(1)} = {\mathbb{C}}^{hom,MT}$). The Mori-Tanaka estimate ${\mathbb{C}}^{hom,MT}$ of the configuration at $n = 1$ can be obtained from (12) with ${\mathbb{C}}^{0} = {{\mathbb{c}}}_m$ and $r \in \{ ic,m\}$. We summarize the algorithm for cascade levels $n = 1$ and $n = 2$:

41a $$\begin{aligned}&\begin{aligned}&\text {n = 1:} \;\;\;\;\; {\mathbb{C}}^{hom,(n-1)} = {{\mathbb{c}}}_m \\&{\mathbb{C}}^{hom,(1)} = {\mathbb{C}}^{hom,MT} \\&\quad = \Big [\phi _m \; {{\mathbb{c}}}_m + \phi _{ic} \; {{\mathbb{c}}}_{ic}:[{\mathbb{I}} + {\mathbb{P}}_{ic}^{m}: ({{\mathbb{c}}}_{ic} - {{\mathbb{c}}}_{m})]^{-1}\Big ]: \\&\qquad \Big [\phi _{m}\; {\mathbb{I}} + \phi _{ic} [{\mathbb{I}} + {\mathbb{P}}_{ic}^{m}:({{\mathbb{c}}}_{ic} -{{\mathbb{c}}}_{m})]^{-1} \Big ]^{-1} , \end{aligned} \end{aligned}$$

41b $$\begin{aligned}&\begin{aligned}&\text {n = 2:} \;\;\;\;\; {\mathbb{C}}^{hom,(n-1)} = {\mathbb{C}}^{hom,(1)} \\&{\mathbb{C}}^{hom,(2)} \\&\quad = \Big [\phi _m \; {\mathbb{C}}^{hom,(1)} + \phi _{ic} \; {{\mathbb{c}}}_{ic}: \\&\qquad \quad [{\mathbb{I}} + {\mathbb{P}}_{ic}^{hom,(1)}: ({{\mathbb{c}}}_{ic} - {\mathbb{C}}^{hom,(1)})]^{-1}\Big ]: \\&\qquad \Big [\phi _{m}\; {\mathbb{I}} + \phi _{ic} [{\mathbb{I}} + {\mathbb{P}}_{ic}^{hom,(1)}: ({{\mathbb{c}}}_{ic} -{\mathbb{C}}^{hom,(1)})]^{-1} \Big ]^{-1}. \end{aligned} \end{aligned}$$

The cascade level *n* can be thought of as a reflection of the connectivity of the inclusion phase. $n = 1$ represents the isolated inclusions in a continuous matrix (the Mori-Tanaka estimate). $n \rightarrow \infty$ represents a completely intermixed inclusion phase (the self-consistent estimate). This feature enables CCM to predict homogenization estimates for a large range of volume fractions of the inclusion phase with different microstructure quality.

### B. Some more details on model input data for oats

The micromechanics model for homogenized stiffness and limit states developed in Sect. 3 requires “universal” material properties of base materials cellulose, hemicellulose and lignin, and their hierarchical composition according to Fig 5. The “universal” mechanical properties of constituents are listed in Table 2. Cousins found that the stiffness of lignin and hemicellulose decreases with increasing moisture content (Cousins 1978, 1976), and reported the relations with the relative humidity. The graphs read 67% reduction in Young‘s modulus of both lignin and hemicellulose at the relative humidity of 70%, the average relative humidity in St. Paul. We assume proportional reduction in the shear strength of lignin too. The crystallinity index (CI), which quantifies the fraction of crystalline cellulose in relation to the volume of the whole cellulose, has been predicted experimentally using X-ray diffraction for oats as $CI=0.44$ (Espinosa et al. 2017). The volume fraction of the constituents in the cell wall material RVEs in Fig. 5 can easily be calculated from chemical composition data listed in Table 1 (Gangwar and Schillinger 2019).

The volume fractions of phases in all other RVEs in Fig. 5 are the other required inputs for the model. We derive the volume fractions with the help of microimages reported in Sect. 2. The data are reported in Table 4.

**Table 4:** Measured model parameters phases

Variety	$\phi ^{par}_{wall}$	$\phi ^{fib^{*}}_{wall}$	$\phi _{fib}$	$\phi _{vb}$	$\phi ^{shell^{*}}_{wall}$	$t/t_{shell}$
Gopher	0.18	0.50	0.82	0.16	0.40	0.14
Reins	0.15	0.50	0.85	0.13	0.40	0.15
ND021052	0.17	0.50	0.80	0.11	0.40	0.16
IL07-8721	0.20	0.50	0.90	0.13	0.40	0.15

*Measured from TEM images of oats of the Gopher variety. In absence of imaging data, we assume that other varieties share the same volume fractions

### C. Four-point bending test results for oat specimens

In Table 5, we also summarize the geometric measurements, the linear-elastic slope $({\varDelta }P/ {\varDelta }\delta )_{\textit{exp}}$, and the failure load $P^{\textit{max}}_{\textit{exp}}$ for each four-point bending test specimen. Please see Sect. 4 for further details.

**Table 5:** Linear-elastic slope and failure load: four-point bending test results vs model predictions along with geometric measurements

Variety	a (mm)	b (mm)	t (mm)	$({\varDelta }P/ {\varDelta }\delta )_{exp}$	$P^{max}_{exp}$	$({\varDelta }P/ {\varDelta }\delta )_{est}$	$P^{max}_{est}$
Gopher	1.77	1.60	0.36	6.5	5.7	5.0	5.4
Gopher	1.52	1.31	0.38	4.8	5.8	3.8	4.8
Gopher	1.45	1.42	0.60	7.3	5.9	6.2	8.5
Gopher	1.75	1.56	0.31	4.5	4.9	3.7	3.9
Gopher	1.66	1.52	0.30	5.6	4.6	3.5	3.6
Gopher	1.54	1.50	0.43	6.5	5.2	5.5	6.7
Reins	2.12	1.96	0.60	6.3	10.3	8.7	11.2
Reins	2.04	1.89	0.44	8.7	8.6	5.9	6.9
Reins	1.67	1.51	0.64	8.4	8.9	6.0	8.4
Reins	1.74	1.63	0.39	8.5	7.6	4.0	4.6
Reins	1.97	1.86	0.58	8.4	11.3	7.9	10.1
Reins	1.76	1.74	0.46	6.7	6.6	5.4	6.6
ND021052	1.35	1.34	0.41	5.9	5.8	2.7	4.2
ND021052	1.83	1.79	0.40	4.9	5.1	3.9	5.3
ND021052	1.67	1.56	0.51	6.5	6.1	4.4	6.6
ND021052	1.93	1.86	0.35	4.7	5.2	3.7	4.8
ND021052	1.83	1.71	0.88	7.5	11.7	8.4	14.7
ND021052	1.80	1.70	0.39	2.8	3.7	3.7	4.8
IL07-8721	1.50	1.47	0.57	11.2	11.3	5.5	8.1
IL07-8721	1.79	1.56	0.98	15.8	16.7	10.1	15.8
IL07-8721	1.68	1.54	0.83	8.4	11.1	8.3	12.5
IL07-8721	1.74	1.71	0.46	8.0	7.2	6.3	7.7
IL07-8721	1.84	1.74	0.46	5.9	6.5	6.4	7.8
IL07-8721	1.79	1.69	0.45	5.5	5.8	6.1	7.6

Refer to Fig. 6a for the meaning of **a**, **b**, and **t**
