# Correction to: Multiscale characterization and micromechanical modeling of crop stem materials

**DOI:** 10.1007/s10237-021-01490-0

**Published:** 2021-07-23

**Authors:** Tarun Gangwar, D. Jo Heuschele, George Annor, Alex Fok, Kevin P. Smith, Dominik Schillinger

**Affiliations:** 1 Department of Civil, Environmental, and Geo- Engineering, University of Minnesota, Twin Cities, USA; 2 Department of Agronomy and Plant Genetics, University of Minnesota, Twin Cities, USA; 3 Department of Food Science and Nutrition, University of Minnesota, Twin Cities, USA; 4 Minnesota Dental Research Center for Biomaterials and Biomechanics, University of Minnesota, Twin Cities, USA

## Correction to: Biomechanics and Modeling in Mechanobiology (2021) 20:69–91 10.1007/s10237-020-01369-6

The article “Multiscale characterization and micromechanical modeling of crop stem materials”, written by Tarun Gangwar, D. Jo Heuschele, George Annor, Alex Fok, Kevin P. Smith, Dominik Schillinger, was originally published Online First without Open Access. After publication in volume 20, issue 1, pages 69–91, the author decided to opt for Open Choice and to make the article an Open Access publication. Therefore, the copyright of the article has been changed to ©The Author(s) 2021, and the article is forthwith distributed under the terms of the Creative Commons Attribution 4.0 International License, which permits use, sharing, adaptation, distribution and reproduction in any medium or format, as long as you give appropriate credit to the original author(s) and the source, provide a link to the Creative Commons licence, and indicate if changes were made. The images or other third-party material in this article is included in the article’s Creative Commons licence, unless indicated otherwise in a credit line to the material. If material is not included in the article’s Creative Commons licence and your intended use is not permitted by statutory regulation or exceeds the permitted use, you will need to obtain permission directly from the copyright holder. To view a copy of this licence, visit http://creativecommons.org/licenses/by/4.0.

The original article has been corrected.

